# Second victim experiences of healthcare providers after adverse events: A cross-sectional study

**DOI:** 10.4102/hsag.v27i0.1858

**Published:** 2022-08-29

**Authors:** Le Crenis Mathebula, Celia J. Filmalter, Joyce Jordaan, Tanya Heyns

**Affiliations:** 1 School of Healthcare Sciences, Faculty of Health Sciences, University of Pretoria, Pretoria, South Africa; 2 Department of Statistics, Faculty of Informatics, University of Pretoria, Pretoria, South Africa

**Keywords:** adverse events, healthcare professionals, healthcare providers, patient safety, second victims

## Abstract

**Background:**

Adverse events in healthcare are inevitable as most treatments and investigations have the potential to cause harm. Healthcare providers often witness or are involved in adverse events, putting them at risk of becoming second victims, which may further impact patient safety.

**Aim:**

The researchers report on the physical and psychological symptoms experienced by healthcare providers following adverse events during patient care as well as their perceptions of the quality of support received and the desired forms of support following adverse events.

**Setting:**

A single secondary public hospital in the Limpopo province, South Africa.

**Methods:**

Using total population sampling, healthcare providers were invited to anonymously participate in a cross-sectional survey using the Second Victim Experience and Support questionnaire to assess experiences after adverse events and desired forms of support.

**Results:**

Healthcare providers (*N* = 181) experienced more psychological distress (mean = 2.97, standard deviation [SD] = 1.33) than they experienced physical distress. Most healthcare providers relied on non-work-related support (mean = 4.08, SD = 1.19). Healthcare providers reported that adverse events influenced their perceptions of professional self-efficacy (mean = 2.71, SD = 0.94) and mostly desired support in the form of discussing the event with supervisors or managers (mean = 3.72, SD = 1.37).

**Conclusion:**

Healthcare providers in different clinical settings are at risk of suffering second victim effects. Health institutions should offer support to all victims of adverse events.

**Contribution:**

The information offered could enable healthcare management to modify existing practices to a non-punitive style, improve communication and provide better support following adverse events.

## Introduction

In healthcare settings, optimal care is achieved by providing safe, quality care and preventing injuries. In developing countries, eight out of every 100 patients suffer from healthcare-related adverse events (World Health Organization (WHO 2021). Adverse events are defined as unintentional injuries or complications caused by medical interventions and sadly are an inevitable reality (Rafter et al. 2017). In 2004, the WHO initiated worldwide patient safety programmes to address adverse events and promote patient safety (‘Patient Safety’ 2017). Despite these initiatives, adverse events persist in the healthcare environment and many are unrelated to patients’ underlying conditions (Sanchez et al. 2017). The first victims of adverse events are the patients who are harmed and traumatised, and their families (Mira et al. 2017). The patient may end up with long-term complications or morbidities, causing them to lose their ability to function normally (Urden, Stacy & Lough 2017). Families may have to assume the burden of caring for their loved one with morbidities or they may even suffer the death of a loved one following an adverse event (Busch et al. 2020a). Adverse events may also lead to patients and families being afraid of and distrusting healthcare providers (Mira et al. 2017). Most patient safety programmes emphasise the well-being of patients and their families and help them to deal with the aftermath of adverse events (Urden et al. 2017).

When patients suffer adverse events, healthcare providers may also be harmed and traumatised either directly or indirectly and become second victims (Busch et al. 2020b). Healthcare providers are ethically bound to ‘do no harm’ to their patients and to society. When adverse events occur, healthcare providers may become distressed if they were involved, witnessed or failed to prevent an adverse event. They are also expected to care for patients, and these expectations put them under pressure, aggravating distress, resulting in the second victim experience (Mira et al. 2017).

The term ‘second victim’ was first used by Wu (2000), when referring to healthcare providers who were traumatised by being involved in patients’ adverse events. Second victims often present with feelings of guilt, frustration, fatigue and insomnia (Han et al. 2017). Some healthcare providers become hypervigilant, and others withdraw during procedures that remind them of the previously experienced adverse event (Ozeke et al. 2019). To cope with these feelings, healthcare providers may engage in destructive behaviours such as using drugs, nicotine, and alcohol, behaviours that are consistent with post-traumatic stress disorder (Baas et al. 2018; Busch et al. 2020b). As second victims, healthcare providers who experience adverse events may even contemplate switching careers because of reduced job satisfaction (Kable, Kelly & Adams 2018). If not managed, second victim effects may damage healthcare providers’ psychological and physical health, further compromising patient safety (Brannon 2020). Health care providers thus require support in dealing with adverse events.

In healthcare settings, second victim support has been given limited attention (Lane et al. 2018). Currently, many healthcare institutions have a blaming and punitive culture where adverse events are investigated and culpability is assigned without considering the factors that precipitated the adverse event (Han et al. 2017). Managing adverse events in this way may exacerbate the burden of being a second victim (Wu et al. 2020). Ideally, healthcare institutions should ensure that structures are in place to support second victims (Lane et al. 2018). There is a growing body of evidence suggesting that second victims should be offered timely and easily accessible support to help cope with the trauma of adverse events (Mjadu & Jarvis 2018; Wu et al. 2020). Timely support promotes staff well-being, retention of staff members and readiness to give quality care (Kable et al. 2018). According to the World Health Organization (2021), 134 million adverse events are reported in hospitals from low- and middle-income countries, implying that South Africa has a large burden of adverse events (Nydoo et al. 2020). The experiences of healthcare providers as second victims have not been widely investigated. Exploring and understanding the experiences and support needs of healthcare providers could serve as a roadmap for developing policies and stuctures to support second victims in healthcare settings (Chan et al. 2018). In this article, the authors report on healthcare providers’ experiences of adverse events in a South African hospital, the associated physical and psychological symptoms experienced, the quality of support received and the desired support expected after experiencing an adverse event.

## Methods

### Study design and participants

The authors conducted this cross-sectional study from August 2020 to September 2020 in a 316-bed secondary public hospital in the Limpopo province. The hospital serves 131 rural villages in a municipal area with an estimated population of 212 701. The hospital staff complement for 2020 totalled 826, including healthcare providers, allied healthcare providers and administrative personnel.

The authors adopted total sampling, inviting all healthcare providers delivering direct healthcare to patients (*n* = 593) who have been exposed to an adverse event to participate in the study. In alphabetical order, healthcare providers included antiretroviral counsellors and testers, dieticians, dentists and dentist assistants, doctors, forensic pathology workers, nurses, occupational therapists, optometrists, pharmacists, porters, physiotherapists, psychologists, radiographers, and speech and audiology therapists. All participants who volunteered to participate in the study signed an informed consent form.

### Survey questionnaire

We collected data using the Second Victim Experience and Support Tool developed and validated by Burlison et al. (2017). The questionnaire comprised five sections: Section A, demographic data (including age, marital status, years of experience, and clinical unit where participants were placed); Section B, the second victim experience; Section C, the support received, and Section D, the professional self-efficacy. The questionnaire has 10 survey items, namely, psychological distress (embarrassment, fear and remorse), physical distress (exhaustion, sleep disturbances, nausea and loss of appetite), colleagues support, supervisor support, institutional support, non-work-related support, professional efficacy, turnover intentions, absenteeism and desired form of support (Burlison et al. 2017). The participants were asked to indicate the extent to which they agree or disagree with each statement, using a five-point Likert scale, where 1 is strongly disagree, 2 disagree, 3 neutral, 4 agree and 5 strongly agree. Section E measured the desired form of support on a five-point Likert scale. Using Cronbach’s α scores, the reliability of the original tool was measured as 0.61 (co-worker support) and 0.87 (supervisor support) and scores on all survey dimensions and outcome variables were greater than 0.70, with the exception of colleague support and organisational support (Burlison et al. 2017).

### Data collection

Upon ethical approval of the study, the researchers conducted a pilot test on five healthcare providers. The questionnaire did not require adjustments. The first author then visited all the clinical units in the selected hospital. The heads of each unit received a information leaflet and copies of the questionnaire. The heads of departments volunteered to distribute the questionnaires to prospective participants and briefly inform them about the study. The participants voluntarily read through the information leaflet and completed the questionnaire. Participants completed the anonymous questionnaires and put them into a tamper-proof box in the unit managers’ office. The box was collected by the first author 1 week later.

### Data analysis

The data were captured in Microsoft Excel sheets and analysed using IBM SPSS statistics version 26. The seven items measuring the desired forms of support were analysed using frequencies and descriptive statistics. Three items were reverse-coded and Cronbach’s alphas were computed for each of the sub-scales. Mean scores were calculated for each of the sub-scales and frequencies, and descriptive statistics produced. The medians of the sub-scales were compared across three main occupation groups (nurses, doctors and allied healthcare providers) using Kruskal-Wallis tests (see Table 1).

**TABLE 1:** Second victim-related distress of healthcare providers in a public hospital.

Survey items	Cronbach’s alpha	General		Nurses		Doctors		Allied Healthcare providers	
		Mean	Standard deviation	Mean	Standard deviation	Mean	Standard deviation	Mean	Standard deviation
1. Psychological distress	0.902	2.97	1.33	2.99	1.35	3.4	1.07	2.65	1.3
2. Physical distress	0.853	2.52	1.20	2.53	1.20	2.68	1.10	2.39	1.28
3. Colleague’s support	0.756	3.30	1.06	3.30	1.06	3.5	0.69	3.16	1.24
4. Supervisor’s support	0.850	3.43	0.99	3.32	0.92	3.8	0.85	3.51	1.22
5. Institution support	0.787	2.94	0.88	2.96	0.85	2.8	0.72	2.99	1.06
6. Not work-related support	0.868	4.08	1.19	4.11	1.17	4.29	0.85	3.87	1.39
7. Professional self-efficacy	0.763	2.71	0.94	2.76	0.94	2.83	0.81	2.51	0.98
8. Turnover intentions	0.777	2.56	1.40	2.61	1.43	2.64	1.27	2.37	1.41
9. Absenteeism	0.730	2.08	1.26	2.02	1.19	1.75	1.09	2.48	1.50

### Ethical considerations

Ethical approval was obtained from the Faculty of Health Research Ethics Committee of the University of Pretoria (No: 287/2020) and the Limpopo Department of Health South Africa (L-2020-05-09). The Chief Executive Officer of the selected hospital also approved the study. The first author visited all the heads of departments in the hospital to inform them about the study.

## Results

### Demographic information

Of the eligible healthcare providers involved in direct patient care (*n* = 593), 181 completed the questionnaire, representing a response rate of 30.5%. The healthcare providers included 118 nurses, 24 medical doctors and 39 allied healthcare providers. Allied healthcare providers included antiretroviral counsellors and testers (*n* = 5), dieticians (*n* = 2), dentists (*n* = 5), dental assistants (*n* = 2), forensic pathology workers (*n* = 2), porters (*n* = 4), an occupational therapist (*n* = 1), an optometrist (*n* = 1), pharmacists (*n* = 8), physiotherapists (*n* = 4), psychologists (*n* = 3), radiographers (*n* = 1) and a speech and audiology worker (*n* = 1).

The participants were on average 42.35 years old (standard deviation [SD] = 9.825 years); 20.4% (*n* = 37) of participants did not specify their age. The participants comprised 83.4% (*n* = 151) women and 16.6% (*n* = 30) men. Of these participants, 43.6% (*n* = 79) were married and 56.4% (*n* = 102) stated that they were single. On average, participants had 13.5 (SD = 8.79) years’ work experience, with 29.8% (*n* = 54) working in the same healthcare institution for 11–15 years.

### Second victim experience

The participants’ second victim experience, related distress and support were categorised based on the following: nurses, doctors and allied healthcare providers, which are captured in Table 1.

This group of healthcare providers (see Table 1) more often experienced psychological distress (2.97) than physical distress (2.52) after experiencing adverse events. Doctors experienced more distress (3.4) when compared to nurses (2.99) and allied healthcare workers (2.65); however, the difference was not statistically significant (*p* = 0.69).

Adverse events affected healthcare providers’ professional self-efficacy the most (2.71) followed by turnover intentions (2.56) and absenteeism (2.08). In this study, healthcare providers experienced professional doubt following adverse events, which affected them more than influencing their desire to leave the profession or being absent from work.

Healthcare providers were supported largely by non-work-related structures (4.08), followed by supervisor support (3.43), support from colleagues (3.30) and support from their institution (2.94). In this setting, institutional support was limited and non-work-related support was regarded as effective. Table 2 presents the support options preferred by participants.

**TABLE 2:** Support options preferred by participants.

Survey item	Agree		Disagree		Neutral		Mean	Standard deviation
	Count	%	Count	%	Count	%		
The ability to immediately take away from my unit for a little while	55	30.9	85	47.8	38	21.3	2.74	1.52
A specified peaceful location that is available to recover and recompose after one of these types of events	66	36.5	76	42.0	39	21.5	2.89	1.48
A respected peer to discuss the detail of what happened	104	57.5	41	22.7	36	19.9	3.5	1.52
An employee-assisted programme that can provide free counselling to employees outside of work	107	59.1	47	26.0	27	14.9	3.55	1.52
A discussion with the manager or supervisor about the incident	117	65.5	37	20.6	26	14.4	3.72	1.37
The opportunity to schedule a time with a counsellor at my hospital to discuss the event	99	55.0	45	25.0	36	20.0	3.49	1.45
A confidential way to get in touch with someone 24 h a day to discuss how my experience may be affecting me	94	52.8	63	35.4	21	11.8	3.37	1.56

*Source*: Burlison, J.D., Scott, S.D., Browne, E.K., Thompson, S.G. & Hoffman, J.M., 2017, ‘The second victim experience and support tool: Validation of an organizational resource for assessing second victim effects and the quality of support resources’, *Journal of Patient Safety* 13(2), 93–102. https://doi.org/10.1097/PTS.0000000000000129

Healthcare providers mostly desired support in the form of needing to discuss the event with a supervisor or manager (agreed 65.5%, neutral 14.4%, disagreed 20.6%; the mean score was 3.72 with a SD of 1.37. Healthcare providers did not agree that taking time off work was desirable (disagreed 47.8%, neutral 21.3%, agreed 30.9%; the mean score was 2.74 with a SD of 1.52).

## Discussion

A significant number of healthcare providers, from different health specialties, experienced psychological distress following an adverse event. The psychological distress was more pronounced than any physical distress. These findings are similar to other studies describing the experience of healthcare providers on adverse events (Kerkman et al. 2019; Wu et al. 2020). Kerkman et al. (2019) maintained that traumatic events are experienced the same way regardless of setting, personality, working conditions or environmental factors, indicating that trauma is a subjective experience. As second victims, healthcare providers experience psychological distress regardless of the healthcare setting and their position.

The doctors reported psychological distress more, relative to nursing personnel and allied health workers. Doctors, as senior healthcare providers, may experience a greater sense of self-identified personal responsibility, which may influence their individual experience and degree of distress (Finney et al. 2021; Nydoo et al. 2020). Doctors are more likely to hold themselves personally responsible for patients, and feel responsible for any adverse events. Therefore, it is very important to assess their psychological needs and provide support.

The healthcare providers rarely experienced physical distress relative to psychological distress. Physical symptoms may depend on the intensity of psychological symptoms and may be slow to manifest. Existing literature suggests that physical symptoms may differ depending on the type of profession, experience and the extent of the psychological impact or trauma (Kobe et al. 2019; Rodger & Atwal 2018).

To mitigate or handle the effects of stress after a traumatic event, second victims require effective psychosocial support (Burlison et al. 2017). The healthcare providers most often turned to non-work-related support to help them deal with trauma following an adverse event. Healthcare providers may seek support from non-work-related sources because they want to avoid judgement and require a sympathetic ear. The friends and family of second victims do not always fully comprehend the responsibilities and ethical codes regarding healthcare provision and are likely to be more sympathetic, which is what second victims want to experience immediately after experiencing a traumatic event (Mahmoud, Hussain & Salim 2018). Second victims may prefer non-work-related sources of support because friends and family may not fully understand the profession, and thus be spared the full implications of the adverse event (Mok et al. 2020).

Second victims require adequate emotional support from their supervisors. This support depends on the quality of the relationship between the second victim and the supervisor, with poor relationships impeding the second victims’ ability to share, process and cope with their emotions (Kubheka et al. 2020). Participants were less likely to receive support from their supervisors relative to receiving support from outside sources. This could be due to second victims perceiving that supervisors have a conflict of interest between providing support and investigating the event (Kubheka et al. 2020). Informal colleague support was found to buffer the second victim experience and it is thus a desired form of support (Winning et al. 2018).

In South Africa, most health institutions offer Employee Assistant Programmes, to support their employees (Mjadu & Jarvis 2018). The findings of this study show that healthcare providers did not opt for institutional support after adverse events. Second victims may not trust institutional support structures or may not accept that they need help, hampering their requests for support (Lane et al. 2018). Alternatively, second victims may not utilise institutional support because they feel that organisations do not know what they go through after an event (Nydoo et al. 2020).

Healthcare providers who experienced adverse events were more likely to report feelings of low professional self-efficacy relative to turnover intentions and absenteeism. It is likely that experiencing low professional self-efficacy for an extended period of time may lead to an accumulation of stress that ultimately leads to high turnover intentions (Huang et al. 2020; Mok et al. 2020).

Despite not readily receiving support from supervisors, healthcare providers reported a need to be able to discuss adverse events with their supervisors or managers. Being able to discuss these events with managers and supervisors manifests as being valued and cared for, helping healthcare providers move past adverse events (Stone 2020). Institutions need to encourage a blame-free culture of dealing with adverse events to realise healthcare providers’ desires of being able to interact with their supervisors. Burlison et al. (2017) also reported that support from managers could effectively reduce the distress experienced after adverse events. Supervisors and managers should avoid attributing errors to individuals for or finding culpability with healthcare providers (Kubheka et al. 2020; Zhang et al. 2019).

## Implication for nursing

Adverse events are an unintentional but unavoidable reality in the healthcare environment which increases the risk of healthcare providers becoming second victims. Awareness of the potential impact of trauma on healthcare providers as second victims may help to mitigate the long-term effect of adverse events. While doctors, as responsible healthcare providers, may suffer the greatest degree of distress following an adverse event, nursing supervisors may be ideally placed to offer much needed awareness and support. Nurses in leadership positions ought to be trained to identify and promote support for second victims of adverse events. They should be trained to focus on improving relationships between healthcare providers and supervisors. Institutions should develop programmes and procedures for second victims in dealing with adverse events, especially for staff who are directly involved with patient care.

## Limitations and strengths

A validated tool to assess how healthcare providers dealt with adverse events and which support was needed to help them deal with adverse events was used. However, no data were collected regarding an existing policy to deal with second victims. The healthcare providers represented participants from a variety of clinical settings, including those where there was a high potential for adverse events. This study was limited because it took place during the coronavirus disease 2019 (COVID-19) lockdown in South Africa where healthcare providers were inundated with dealing with a pandemic and ever-changing practice. In addition, the data were collected over a restricted time period, introducing the potential for recall bias. The findings are limited to a selected hospital in South Africa and therefore may limit the generalisation, and the type and timing of adverse events were not investigated.

## Conclusion

All healthcare providers take an oath to ‘do no harm’. While healthcare providers strive to meet this ethical expectation, unexpected harm still occurs in the healthcare environment. When an adverse event occurs, the first victim is the patient, and their family, with healthcare providers often being the second victims. Institutions are obliged to support all the victims affected by the adverse event. Good support may minimise effects, such as a loss of confidence, which is important for healthcare providers. Lack of support may increase the duration of negative effects, which may ultimately lead to healthcare providers desiring a change of occupation. This study revealed that healthcare providers in the Limpopo province experience adverse events and are second victims. These healthcare providers need timely support to be able to perform optimally.
